# *Mycobacterium bovis* in Burkina Faso: Epidemiologic and Genetic Links between Human and Cattle Isolates

**DOI:** 10.1371/journal.pntd.0003142

**Published:** 2014-10-02

**Authors:** Adama Sanou, Zekiba Tarnagda, Estelle Kanyala, Dezemon Zingué, Moumini Nouctara, Zakaria Ganamé, Adjima Combary, Hervé Hien, Mathurin Dembele, Antoinette Kabore, Nicolas Meda, Philippe Van de Perre, Dorine Neveu, Anne Laure Bañuls, Sylvain Godreuil

**Affiliations:** 1 MIVEGEC, UMR IRD 224-CNRS 5290-Montpellier Universités 1 et 2, Montpellier, France; 2 Centre Muraz, Bobo-Dioulasso, Burkina Faso; 3 Institut de Recherche en Sciences de la Santé (IRSS), Direction Régionale de l'Ouest (DRO), Bobo Dioulasso, Burkina Faso; 4 Université de Ouagadougou, Ouagadougou, Burkina Faso; 5 Programme National Tuberculose, Ouagadougou, Burkina Faso; 6 Centre Hospitalier Régional Universitaire (CHRU) de Montpellier, Département de Bactériologie-Virologie, Montpellier, France; 7 Université Montpellier 1, Montpellier, France; 8 INSERM U 1058, Infection by HIV and by Agents with Mucocutaneous Tropism: From Pathogenesis to Prevention, Montpellier, France; University of California, San Diego, United States of America

## Abstract

**Background:**

In sub-Saharan Africa, bovine tuberculosis (bTB) is a potential hazard for animals and humans health. The goal of this study was to improve our understanding of bTB epidemiology in Burkina Faso and especially *Mycobacterium bovis* transmission within and between the bovine and human populations.

**Methodology/principal findings:**

Twenty six *M. bovis* strains were isolated from 101 cattle carcasses with suspected bTB lesions during routine meat inspections at the Bobo Dioulasso and Ouagadougou slaughterhouses. In addition, 7 *M. bovis* strains were isolated from 576 patients with pulmonary tuberculosis. Spoligotyping, RDAf1 deletion and MIRU-VNTR typing were used for strains genotyping. The isolation of *M. bovis* strains was confirmed by spoligotyping and 12 spoligotype signatures were detected. Together, the spoligotyping and MIRU-VNTR data allowed grouping the 33 *M. bovis* isolates in seven clusters including isolates exclusively from cattle (5) or humans (1) or from both (1). Moreover, these data (genetic analyses and phenetic tree) showed that the *M. bovis* isolates belonged to the African 1 (Af1) clonal complex (81.8%) and the putative African 5 (Af5) clonal complex (18.2%), in agreement with the results of RDAf1 deletion typing.

**Conclusions/Significance:**

This is the first detailed molecular characterization of *M. bovis* strains from humans and cattle in Burkina Faso. The distribution of the two Af1 and putative Af5 clonal complexes is comparable to what has been reported in neighbouring countries. Furthermore, the strain genetic profiles suggest that *M. bovis* circulates across the borders and that the Burkina Faso strains originate from different countries, but have a country-specific evolution. The genetic characterization suggests that, currently, *M. bovis* transmission occurs mainly between cattle, occasionally between cattle and humans and potentially between humans. This study emphasizes the bTB risk in cattle but also in humans and the difficulty to set up proper disease control strategies in Burkina Faso.

## Author Summary

Bovine tuberculosis is an infectious disease caused by *Mycobacterium bovis* in livestock and wild animals. Humans can acquire this germ by aerogenous route when in close contact with infected animals, or by consuming unpasteurized dairy products from infected animals and also through the skin when handling infected carcasses. For the present study in Burkina Faso, *M. bovis* strains were collected from slaughtered animals during routine veterinarian inspection at the slaughterhouses of Bobo Dioulasso and Ouagadougou and also from patients with suspected pulmonary tuberculosis. The isolates were genetically characterized using three techniques: spoligotyping, MIRU-VNTR and RDAf1 deletion analysis. Our results highlight two aspects of *M. bovis* epidemiology that are crucial for disease control: i) *M. bovis* circulates between Burkina Faso and its neighbouring countries and ii) *M. bovis* is transmitted mainly between cattle, but also between cattle and humans, and potentially between humans. This study stresses the need to develop an efficient strategy to control *M. bovis* transmission, but also the difficulty to implement control measures because of the complex epidemiology of bovine tuberculosis in Burkina Faso.

## Introduction

*Mycobacterium bovis* is the causative agent of bovine tuberculosis (bTB) in a broad spectrum of hosts, such as cattle, goats, sheep and wild animals to which it can be transmitted through the oral or respiratory route [1]. Humans also can acquire *M. bovis* generally through the aerogenous route when in close contact with infected animals, but also by consuming unpasteurized dairy products from infected animals and through the skin when handling infected carcasses [2], [3], [4].

In sub-Saharan Africa, bTB is a serious problem for livestock production but also a health risk for humans as most human populations live in close contact with domestic animals in which the disease is highly prevalent and imperfectly controlled [5]. Therefore, bTB has a deleterious economic burden, although this has not been quantified in Africa as yet [5], [6]. In Burkina Faso, little is known about bTB epidemiology and national strategies for disease control are almost non-existent [7]. Tuberculin testing of livestock is not routinely performed and bTB screening is limited to visually checking the meat in abattoirs. Despite the high prevalence of bTB in cattle and the presence of *M. bovis* in 26.5% of unpasteurized milk samples, the zoonotic transmission of bTB is also poorly known [8]. In Burkina Faso, cattle breeding relies mainly on extensive transhumance and is the prerogative of few ethnic groups [7]. These populations live in close and permanent contact with their livestock and consume raw and unpasteurized dairy products that could favour *M. bovis* transmission [8]. Moreover, in slaughterhouses where cattle are slaughtered, butchers wear minimal protective clothing and handle infected offals with bare hands [8]. These close contacts are an important source of zoonotic transmission [7], [8], [9]. Although culture and species identification of the *M. tuberculosis* complex are not routinely performed in Burkina Faso, previous studies have suggested that *M. bovis* is present in 0.4% to 1.4% of isolates from patients with pulmonary tuberculosis [10], [11].

The main goal of this work was thus to genetically characterize the *M. bovis* population in Burkina Faso in order to improve our understanding of bTB epidemiology and the circulation of *M. bovis* within and between the bovine and human populations in this country. We used spoligotyping and mycobacterial interspersed repetitive units-variable number of tandem repeats (MIRU-VNTR) analysis, because a combination of them is a powerful tool for the genetic characterization of *M. bovis* [12], [13]. We also explored the presence or absence of a specific chromosomal region called RDAf1, which is a region of difference in *M. bovis* [14].

## Materials and Methods

### Human and cattle samples

The tissues showing macroscopic lesions compatible with bTB were collected from slaughtered cattle carcasses during the post-mortem inspection at the slaughterhouses of Ouagadougou and Bobo Dioulasso (the two largest cities of Burkina Faso) between May and October 2011. The cattle slaughtered in these two slaughterhouses are mainly originated from neighbouring villages of Ouagadougou and Bobo Dioulasso, as well as the main areas of cattle production throughout the country and the cattle production system is mainly pastoralism. Samples were collected and transported in sterile containers at 4°C to the Mycobacteria Laboratory of the Muraz Centre (in Bobo Dioulasso) for analysis.

Smear positive sputum specimens of patients with suspected pulmonary TB were collected in the framework of two studies in Burkina Faso: i) a nationwide survey on anti-tuberculosis drug resistance between 2009 and 2011 (funded by the Global Fund); ii) and a regional study in the Hauts Bassins area between 2011 and 2013 (funded by the French National Agency for Research on AIDS and Viral Hepatitis, ANRS 1224 project, “Impact of HIV/*Mycobacterium tuberculosis* co-infection on the dynamics of tuberculosis transmission in Burkina Faso”).

### Isolation and identification of mycobacteria

Bovine tissue samples were processed for mycobacteria isolation following the standard procedures described by the World Organization for Animal Health [15]. Briefly, tissues with tuberculous lesions were dissected into pieces using sterile scissors and forceps, and were then crushed using sterile sea-sand, mortar and pestle. The homogenate of each sample was recovered into 50 ml sterile tube with 10 ml of sterile distilled water, and the obtained solution was homogenized on a vortex mixer for few minutes. After 5 minutes of settling, two milliliters of supernatant were decontaminated with 10 ml of NaOH at 4% according to the protocol described by Petroff's method. Patients' sputum specimens were treated according to the Petroff's method too. The obtained suspensions were inoculated in four Lowenstein-Jensen (LJ) slants, two of which were supplemented with 0.2% of sodium pyruvate. Isolates were identified as mycobacteria and as *M. bovis* species by Ziehl-Neelsen (ZN) staining for Acid Fast Bacilli (AFB) and a conventional biochemical method, previously described by Ledru et al. [11].

### Genotyping

DNA extraction from mycobacteria isolates and high-throughput spoligotyping on Luminex 100 (Luminex Corp., TX) were performed as previously described [16], [17]. The obtained data were compared with those of the international databases SpolDB4.0 [18] and http://www.Mbovis.org [19]. The new spoligotypes were submitted to the http://www.Mbovis.org database, and new SB numbers (spoligotype codes) were assigned accordingly.

Human and cattle isolates were also genotyped by PCR amplification of 26 MIRU-VNTR loci: ETR A, B, C, D, E; QUB-11a, 11b, 26, 4156, 3232; MIRU 2, 10, 16, 20, 23, 24, 26, 27, 39, 40 and Mtub 04, 21, 29, 30, 34, 39 [20], [21]. We used multiplex PCR and capillary electrophoresis-based sequencers (ABI 3730-XL), as previously described [22]. PCR fragment sizing and assignment of the different MIRU-VNTR alleles were done using Genemapper, version 4.0 (PE Applied Biosystems). The results for each of the 26 loci were combined into 26-digit allelic profiles [22].

Finally, the presence of the RDAf1 deletion was determined using a multiplex PCR method with a set of three primers followed by agarose gel electrophoresis according to Müller et al. [14].

### Genetic diversity and population structure analyses

Several diversity indices, including the genotypic diversity (*Gd* = the number of different genotypes divided by the total number of samples using the combination of MIRU-VNTR and Spoligotyping data), the allelic diversity per locus and the mean genetic diversity (*H*_s_) were calculated. The population structure was explored by estimating the *F*_st_ (index of genetic differentiation between samples) value (0 = no differentiation and 1 = fixation of alternative alleles). The allelic diversity, the *H*_s_ and the *F*_st_ were calculated using F-STAT, version 2.9.3 with the 26 MIRU-VNTR loci [23].

### Phenetic tree and statistical analyses

Genetic relationships among isolates were built with the UPGMA (unweighted pair group method with arithmetic average) and NJ (Neighbour Joining) clustering methods using the MIRU-VNTR and spoligotyping data. The Phylip and Populations packages [24] were used for tree elaboration based on the Nei's distance, and Treedyn for tree visualization and annotation [25].

### Ethical considerations

The recruitment of human patients and the collection of bovine samples were done according to protocols approved by the Ethics Committee for Health Research in Burkina Faso (2007-031; June 28, 2009 and 2010-049; 7 July 2010) and by the Ministry of Animal Resources and Fishery. All patients and cattle owners provided written informed consent. The bovine study was conducted according to guidelines recommended by the Government of Burkina Faso (*KITI n° AN VII 114 FP-AGRI-EL portant règlementation de la santé publique vétérinaire au Burkina Faso*).

## Results

### *M. bovis* strains in human and cattle samples

Among the sputum samples collected between 2009 and 2013, the presence of *M. bovis* was biochemically confirmed in 5/269 (1.85%) samples from the nationwide survey and in 2/307 (0.65%) samples from the regional study respectively. Patients originated from cities that were quite distant one from each other (Table 1). Out of the 6 patients (6/7) with available HIV serology, only one (1/6) was HIV-positive.

**Table 1:** Socio-demographic informations about hosts and *M. bovis* isolates.

Strain ID	Host	Geographical location	HIV status^a^	Year of isolation	Number
c1–c7	cattle	Bobo-Dioulasso		2011	7^b^
c8–c26	cattle	Ouagadougou		2011	19^b^
h1	human	Solenzo	HIV-1+	2009	1^c^
h2	human	Ouagadougou	unknown	2010	1^c^
h3	human	Ouagadougou	HIV-	2010	1^c^
h4	human	Koupéla	HIV-	2011	1^c^
h5	human	Bobo-Dioulasso	HIV-	2011	1^c^
h6	human	Bobo-Dioulasso	HIV-	2011	1^d^
h7	human	Bobo-Dioulasso	HIV-	2013	1^d^

a specific to human hosts,

b bovine study,

c nationwide survey,

d regional study.

Among the 1499 cattle carcasses inspected between May and October 2011, suspicious TB lesions were detected in 101 (6.74%) and 48/101 (47.5%) had a mycobacterial culture positive for AFB. Of the 48 strains isolated, 26 were biochemically identified as *M. bovis*. The remaining 22 samples were either nontuberculous mycobacteria (2/22), or *M. africanum* (3/22), or *M. tuberculosis* (2/22), or were contaminated (9/22) or had an insufficient growth (6/22).

### Genetic characterization

**Figure 1:**
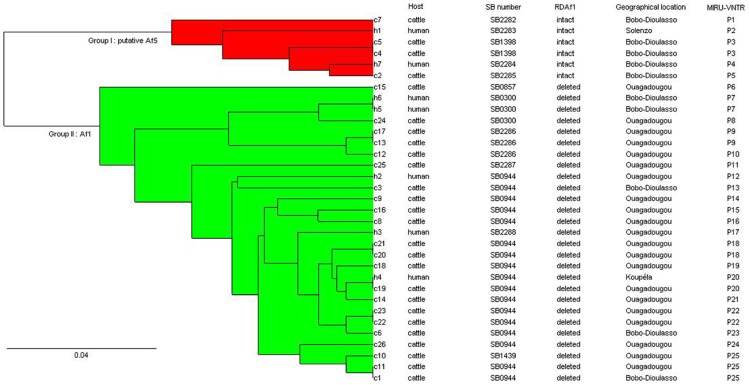
UPGMA tree based on the MIRU-VNTR (26 loci) and spoligotyping data. 1, SB number = name of spoligotype based on http://www.Mbovis.org database nomenclature; 2, RDAf1 = Genomic deletion specific to Af1 clonal complex; 3, The MIRU-VNTR patterns are detailed in table 2.

Spoligotyping confirmed species identification for all 33 *M. bovis* isolates. Among the 12 spoligotype signatures obtained (Table 2), only five (SB1398, SB0300, SB0857, SB0944, SB1439) were already described in the http://www.Mbovis.org database. New codes (SB2282, SB2283, SB2284, SB2285, SB2286, SB2287 and SB2288) have been assigned to the 7 spoligotypes absent in the database. Based on their spoligotype signature, 25 isolates (75.8%) were clustered in 4 groups. The largest cluster included 17 strains bearing the SB0944 spoligotype profile; two clusters had only three strains each (SB0300 and SB2286 spoligotypes) and the last cluster included two strains with the SB1398 spoligotype. Each of the other eight spoligotype signatures (SB0857, SB1439, SB2282, SB2283, SB2284, SB2285, SB2287 and SB2288) was found in a single *M. bovis* isolate (Fig. 1 and Table 2).

**Table 2:** Spoligotypes, MIRU-VNTR patterns and clonal complex identification of the *M. bovis* strains isolated in Burkina Faso.

SB number	Spoligotype signature^1^	MIRU-VNTR (patterns, codes and number of strains)^2^	Strains	Clone [no (%)]^3^
SB2282	**▪□□□□▪▪▪□▪▪▪▪▪▪□▪▪▪□▪▪▪▪▪▪▪▪▪▪▪▪▪▪□□▪▪□□□□□**	4 2 5 3 3 11 3 5 6 1 2 2 3 2 4 2 7 3 2 2 2 2 5 4 3 2 (P1, n = 1)	c7	Af5 [1 (3%)]
SB2283	**▪□□□□▪▪▪□▪▪▪▪▪▪□▪▪▪▪▪▪▪▪▪▪▪▪▪▪▪▪▪▪□▪▪▪□□□□□**	7 2 5 2 3 12 3 11 7 1 2 2 3 2 6 2 1 3 2 2 2 3 5 3 3 2 (P2, n = 1)	h1	Af5 [1 (3%)]
SB1398	**▪▪□□□▪▪▪□▪▪▪▪▪▪□▪▪▪▪▪▪▪▪▪▪▪▪▪▪▪▪▪▪□□▪▪□□□□□**	7 2 5 3 3 11 3 10 5 1 2 2 3 2 4 2 1 3 2 2 2 3 5 3 3 2 (P3, n = 2)	c4, c5	Af5 [2 (6%)]
SB2284	**▪□□□□▪▪▪□▪▪▪▪▪□□▪▪▪▪▪▪▪▪▪▪▪▪▪▪▪▪▪▪□□▪▪□□□□□**	NA (P4, n = 1)	h7	Af5 [1 (3%)]
SB2285	**▪□□□□▪▪▪□▪▪▪▪▪▪□▪▪▪▪▪▪▪▪▪▪▪▪▪▪▪▪▪▪□□▪▪□□□□□**	7 2 5 3 3 11 3 5 5 1 2 2 3 2 6 2 1 3 2 2 2 3 5 3 3 2 (P5, n = 1)	c2	Af5 [1 (3%)]
SB0857	**▪▪□▪▪▪▪▪□▪▪▪▪▪▪□▪▪▪▪▪▪▪▪▪▪▪▪▪□□□□□□□▪▪□□□□□**	4 5 5 3 3 11 3 1 2 1 2 2 3 2 4 2 5 3 2 2 2 3 5 4 3 2 (P6, n = 1)	c15	Af1 [1 (3%)]
SB0300	**▪▪□▪▪□▪▪□▪▪▪▪▪▪□▪▪▪▪▪▪▪▪▪▪▪▪▪□▪▪▪▪▪▪▪▪□□□□□**	5 4 5 3 2 9 2 1 4 1 2 2 3 2 4 2 6 3 2 2 2 3 5 4 3 2 (P7, n = 2)	h5, h6	Af1 [2 (6%)]
		5 4 5 3 3 9 2 1 4 1 2 2 3 2 4 2 6 3 2 2 2 3 5 4 2 2 (P8, n = 1)	c24	Af1 [1 (3%)]
SB2286	**▪▪□▪▪□▪▪□▪▪▪▪□▪□▪▪▪▪▪▪▪▪▪▪▪▪▪□▪▪▪▪▪▪▪▪□□□□□**	5 5 5 3 3 9 2 1 4 1 2 2 2 2 4 2 5 3 2 2 2 3 5 4 3 2 (P9, n = 2)	c13, c17	Af1 [2 (6%)]
		5 5 5 3 3 10 2 1 4 1 2 2 2 2 4 2 5 3 2 2 2 3 5 4 3 2 (P10, n = 1)	c12	Af1 [1 (3%)]
SB2287	**▪▪□▪▪□□▪□▪▪▪▪▪▪□▪▪▪▪▪▪▪▪▪▪▪▪▪□▪▪▪▪□□▪▪□□□□□**	4 5 5 3 3 10 3 1 3 1 2 2 3 2 4 2 4 3 2 2 2 3 5 4 3 2 (P11, n = 1)	c25	Af1 [1 (3%)]
SB0944	**▪▪□▪▪▪▪▪□▪▪▪▪▪▪□▪▪▪▪▪▪▪▪▪▪▪▪▪□▪▪▪▪▪▪▪▪□□□□□**	3 4 5 3 3 11 3 0 3 1 2 2 3 2 4 2 4 3 2 2 2 1 5 4 3 2 (P12, n = 1)	h2	Af1 [17 (52%)]
		3 6 5 3 3 8 3 1 3 1 2 2 3 2 4 2 3 3 2 2 2 3 5 4 3 2 (P13, n = 1)	c3	
		4 5 5 3 3 11 3 1 2 1 2 2 3 2 4 2 3 3 2 2 2 4 5 4 1 2 (P14, n = 1)	c9	
		4 5 5 3 3 11 3 1 2 1 2 2 3 2 4 2 5 3 2 2 2 2 5 4 3 2 (P15, n = 1)	c16	
		4 5 3 3 3 11 3 1 2 2 2 3 2 4 2 5 3 2 2 2 3 5 4 3 2 (P16, n = 1)	c8	
		4 5 5 3 3 11 3 1 3 1 2 2 3 2 4 2 4 3 2 2 2 3 4 4 3 2 (P18, n = 2)	c21, c20	
		4 5 5 3 3 11 3 1 2 1 2 2 3 2 4 2 4 3 2 2 2 3 5 4 3 2 (P19, n = 1)	c18	
		4 5 5 3 3 11 3 1 3 1 2 2 3 2 4 2 4 3 2 2 2 3 5 4 3 2 (P20, n = 2)	h4, c19	
		4 5 2 3 3 11 3 1 3 1 2 2 3 2 4 2 4 3 2 2 2 3 5 4 3 2 (P21, n = 1)	c14	
		3 5 5 3 3 11 3 1 3 1 2 2 3 2 4 2 4 3 2 2 2 3 5 4 3 2 (P22, n = 2)	c22, c23	
		3 6 5 3 3 11 3 1 3 1 2 2 3 2 4 2 4 3 2 2 2 3 5 4 3 2 (P23, n = 1)	c6	
		4 5 5 3 3 10 3 1 3 2 2 3 2 4 2 4 3 2 2 2 3 5 3 3 2 (P24, n = 1)	c26	
		4 5 5 3 3 10 3 1 2 0 2 2 3 2 4 2 4 3 2 2 2 3 5 4 3 2 (P25, n = 2)	c1, c11	
SB1439	**▪▪□▪▪▪▪▪□▪▪▪▪▪□□▪▪▪▪▪▪▪▪▪▪▪▪▪□▪▪▪▪▪▪▪▪□□□□□**	4 5 5 3 3 10 3 1 2 0 2 2 3 2 4 2 4 3 2 2 2 3 5 4 3 2 (P25, n = 1)	c10	Af1 [1 (3%)]
SB2288	**▪▪□▪▪▪▪▪□▪▪▪▪▪▪□▪▪□▪▪▪▪▪▪▪▪▪▪□▪▪▪▪▪▪▪▪□□□□□**	4 5 5 3 3 11 3 1 4 1 2 2 3 2 4 2 4 3 2 2 2 3 5 4 3 2 (P17, n = 1)	h3	Af1 [1 (3%)]
Total	12 Spoligotype signatures	25 MIRU-VNTR patterns	33 (100%)	Af5 [6 (18.2%)]
				Af1 [27 (81.8%)]

1 **▪**, presence of spacer; **□**, absence of spacer.

2 MIRU-VNTR loci: ETR A, ETR B, ETR C, ETR D, ETR E, QUB-11a, QUB-11b, QUB-3232, QUB-26, QUB-4156, MIRU 2, MIRU 10, MIRU 16, MIRU 20, MIRU 23, MIRU 24, MIRU 26, MIRU 27, MIRU 39, MIRU 40, Mtub 04, Mtub 21, Mtub 29, Mtub 30, Mtub 34, Mtub 39. NA = Not Available.

3 Af1 = African 1 clonal complex, Af5 = putative African 5 clonal complex.

By MIRU-VNTR typing, only one (human sample h7, spoligotype = SB2284, pattern P4) of the 33 isolates could not be genotyped. MIRU-VNTR typing produced 24 distinct patterns (Fig. 1 and Table 2). Fifteen isolates (46.9%) were grouped in seven clusters and the other 17 isolates (53.1%) had a specific pattern each. Moreover analysis of the 26 MIRU-VNTR loci, showed that nine loci were monomorphic in the 32 *M. bovis* strains analysed (see Table 3), indicating the presence of moderate polymorphism. The five most discriminatory loci were ETR A, ETR B, QUB-11a, QUB-26 and MIRU 26. The number of alleles for the MIRU-VNTR loci ranged from 1 to 6, with a mean of 2.5 (Table 3). The combination of spoligotyping and MIRU-VNTR typing revealed that fourteen isolates (42.4%) were grouped in seven clusters and the other 18 isolates (57.6%) had a unique pattern. The seven clusters (14 strains) included isolates from cattle (c4–c5, P3; c17–c13, P9; c20–c21, P18; c22–c23, P22; c1–c11, P25), humans (h5–h6, P7) and from humans and cattle (c19-h4, P20) (see Fig. 1 and Table 2). The MIRU-VNTR pattern P25 was split up in two spoligotypes and these results were confirmed by retesting using the two genotyping methods (Table 2).

**Table 3:** Allelic diversity of the 26 MIRU-VNTR loci in *M. bovis* isolates from humans and livestock in Burkina Faso.*

Locus	Number of alleles			Allelic diversity		
	Global (n = 32)	Af1 (n = 27)	Af5 (n = 5)	Global (n = 32)	Af1 (n = 27)	Af5 (n = 5)
ETR A	4	3	2	0.66	0.6	0.4
ETR B	4	4	2	0.54	0.44	0.4
ETR C	3	3	1	0.12	0.15	0
ETR D	2	1	2	0.06	0	0.4
ETR E	2	2	1	0.12	0.14	0
QUB-11a	5	4	2	0.60	0.63	0.4
QUB-11b	2	2	1	0.31	0.36	0
QUB-3232	5	3	4	0.34	0.15	0.9
QUB-26	6	4	3	0.76	0.70	0.7
QUB-4156	2	2	1	0.20	0.22	0
MIRU 2	1	1	1	0	0	0
MIRU 10	1	1	1	0	0	0
MIRU16	2	2	1	0.18	0.2	0
MIRU 20	1	1	1	0	0	0
MIRU 23	2	1	2	0.12	0	0.6
MIRU 24	1	1	1	0	0	0
MIRU 26	6	5	2	0.71	0.62	0.4
MIRU 27	1	1	1	0	0	0
MIRU 39	1	1	1	0	0	0
MIRU 40	1	1	1	0	0	0
Mtub 04	1	1	1	0	0	0
Mtub 21	4	4	2	0.24	0.21	0.4
Mtub 29	2	2	1	0.12	0.14	0
Mtub 30	2	2	2	0.27	0.07	0.4
Mtub 34	3	3	1	0.12	0.14	0
Mtub 39	1	1	1	0	0	0
Mean	2.5	2.15	1.5	0.21	0.18	0.19

*Excluding one strain of the putative African 5 clonal complex that hasn't MIRU-VNTR data.

Af1 = African 1 clonal complex, Af5 = putative African 5 clonal complex.

The RDAf1 deletion was detected in 27/33 isolates (81.8%). For these samples, as expected, the spoligotyping signatures revealed the absence of spacer 30.

### Phenetic analysis

We obtained comparable trees with UPGMA and NJ methods (data not shown). Only the UPGMA is presented here in order to facilitate the confrontation of tree with the spoligotyping data (Fig. 1). From the tree, we could distinguish two groups (Fig. 1). Group I had six strains (18.2%) that were characterized by the absence of spacers 4 and 5, the presence of spacer 30 in the spoligotype signatures and of the RDAf1 region. This group of strains appeared similar to the one described by Müller et al. [26], provisionally called African 5 (Af5) clonal complex. Even if these strains revealed specific spoligotype signatures as described above, no genomic deletion allowed to characterize this group as a well defined clonal complex [14], [26], [27], [28]. In this study, this group of strains is thus called “putative Af5 clonal complex”. Group II included 27 strains that were assigned to the African 1 (Af1) clonal complex based on two criteria: (i) the absence of spacer 30 in the spoligotype; and (ii) the presence of the specific RDAf1 deletion in the genome (Fig. 1) [14].

### Genetic diversity and population structure of *M. bovis* in Burkina Faso

The mean genetic diversity (*H*_s_) and the genotypic diversity (*Gd*) were respectively 0.187 and 0.79. As expected, the genetic differentiation between Af1 and the putative Af5 strains was high and significant [group II (n = 27) *versus* group I (n = 5); *F*_st_ = 0.35; *p*<0.05]. In addition to the RDAf1 deletion and spoligotype signatures, three MIRU-VNTR loci (QUB-3232, QUB-26 and MIRU 26) allowed assigning the isolates to the Af1 or the putative Af5 clonal complex because the number of repetitions (*n*) is ≥5 for putative Af5 and *n*≤1 for Af1 with QUB-3232 locus, *n*≥5 for putative Af5 and *n*≤4 for Af1 with locus QUB-26, *n* = 7 or 1 for putative Af5, and 1<*n*<7 for Af1 with MIRU 26. Nevertheless, these differences should be confirmed on a larger sample with isolates from different regions.

## Discussion

We present here the first detailed molecular characterization of *M. bovis* strains from humans and cattle in Burkina Faso. The 6.8% prevalence of bTB in cattle recorded in the present study on the basis of observable lesions in carcasses is lower than in previously published works. A 19% prevalence was reported for the Bobo Dioulasso slaughterhouse, fifteen years ago [8]. Nevertheless, our finding is in agreement with results in other Sahelian countries [6], [29], [30], [31]. Despite this still high prevalence of bTB in cattle in our study, the prevalence of *M. bovis* in human TB was low (0.6–1.85%), as that reported in other studies in this country [7], [8], [10], [11]. However, prevalences could have been underestimated because only patients with pulmonary TB were included in the present and previous studies. Classically, *M. bovis* is mainly responsible for extra-pulmonary TB [8]. In any case, the presence of glycerol in LJ medium may have negative impact on the yield of *M. bovis* growth. The use of LJ medium with 0.5% of sodium pyruvate and without glycerol could allow the optimization of this mycobacterial species isolation in Burkina Faso.

In our study, the combination of spoligotyping and RDAf1 deletion analysis showed that all *M. bovis* strains belonged either to the Af1 clonal complex, also found in other countries of West-Central Africa, such as Mali, Cameroon, Nigeria, Chad and Niger [14], [32], or to the putative Af5 clonal complex, also previously described in Mali [26]. In Burkina Faso, the putative Af5 strains are geographically located in the Western Region (Bobo Dioulasso and Solenzo), an area bordering Mali. The presence of common or related genotypes between Burkina Faso and Mali can be explained by the transhumance activities between these countries and by the transit of Malian livestock on their way to the South, for instance to Ghana and Nigeria. Spoligotype signatures belonging to the putative Af5 clonal complex have also been reported in Europe [14], [33]. The putative Af5 clonal complex could have been introduced in these African regions from Europe, possibly via North Africa [26], [33], [34]. In the Af1 clonal complex, the SB0944 spoligotype signature is defined as the most recent common ancestor (progenitor) and is the most frequent pattern within this group (40% in Chad, 46.1% in Nigeria and 62.7% in Cameroon). It was also the most abundant (52%) in our study [14]. The spread of the Af1 clonal complex over this large area of West-Central Africa could be explained by the long distance transhumance for livestock production in the Sahel, mainly practised by the Fulani pastoralists [26]. This points out the difficulty to develop an efficient strategy to stop bTB transmission.

As expected, MIRU-VNTR typing revealed more polymorphisms than spoligotyping with a high genotypic diversity, but a low genetic diversity because the majority of the analysed loci presented a low mean allelic diversity. Despite a significant genetic differentiation, the low number of isolates analyzed in this study does not allow any assumption about the chronological emergence of these two groups of strains. Nevertheless, to explain the predominance of the Af1 clonal complex in many countries, Müller and al. have suggested that Af1 might have a selective advantage compared to the putative Af5 [14].

When we compared the *M. bovis* population of Burkina Faso with the populations from Mali, Chad, Nigeria and Cameroon described in Müller et al. [14], we observed a significant genetic differentiation (data not shown). Studies conducted in Chad and Nigeria showed inter-country variation in terms of discriminatory power of MIRU-VNTR loci. These studies and the present one had eight markers in common and only ETR A and ETR B had very high discriminatory power, while MIRU 2 had a very low or no discriminatory power [12], [35]. On the basis of the polymorphic loci, each country presents a specific pool of genotypes, although they also share several genotypes with the neighbouring countries. As suggested by Müller et al., these different genetic patterns could be explained by specific evolutionary processes (such as genetic drift and/or selection pressure) depending on the ecosystem set after the emergence and spread of Af1 and putative Af5 progenitors in each country [14].

From an epidemiological point of view, despite the absence of reliable and accurate conventional data the finding that specific spoligotype signatures/MIRU-VNTR patterns were shared only by bovine isolates or by bovine and human isolates suggests a recent transmission within the cattle population and between cattle and humans. Moreover, two patients were infected by Af1 strains with the same genotype (spoligotype plus MIRU-VNTR results). The sputum samples from these two patients were processed at the same medical centre (TB Diagnosis and Treatment Centre of Dafra, Bobo Dioulasso) and at the Mycobacteria Laboratory of the Muraz Centre in 2011, but not at the same time, thus excluding intra-laboratory contamination. Nevertheless, the epidemiological link could not be definitively established for this cluster. Different routes of contamination could be responsible for the infection: i) contamination by a common animal or food-borne source; ii) human to human transmission. Indeed inter-human transmission cannot be excluded since intra-familial and community based transmissions of human TB cases due to Af1 strains of *M. bovis* have been already suspected [36], [37].

In conclusion, our study shows that two groups of *M. bovis* circulate in Burkina Faso; a major group belonging to the Af1 clonal complex and a minor group belonging to the putative Af5 clonal complex. Furthermore, the comparison with data from other African regions indicates an inter-country transmission associated with a country-specific evolution. Finally, the clusters suggest current transmission that occurs mainly within cattle populations, less frequently between cattle and humans and possibly between humans. This study points out the difficulty to develop an efficient national control strategy of bTB in Burkina Faso.
